# Association of LMP/TAP Gene Polymorphisms with Tuberculosis Susceptibility in Li Population in China

**DOI:** 10.1371/journal.pone.0033051

**Published:** 2012-03-12

**Authors:** Danmei Wang, Yue Zhou, Limin Ji, Tong He, Feng Lin, Rong Lin, Tangchang Lin, Yanna Mo

**Affiliations:** 1 Laboratory of Human Function, Hainan Medical College, Haikou, Hainan Province, People's Republic of China; 2 Department of Reproductive Immunity, National Research Institute for Family Planning, Beijing, People's Republic of China; 3 Tuberculosis Department, Hainan Center for Disease Control and Prevention, Haikou, Hainan Province, People's Republic of China; 4 Respiratory Department, Hainan People's Hospital of Sanya, Sanya, Hainan Province, People's Republic of China; St. Petersburg Pasteur Institute, Russian Federation

## Abstract

**Background:**

Tuberculosis (TB) is a contagious disease affected by multiple genetic and environmental factors. Several association studies have suggested that cellular immune response is vital for controlling and preventing of tuberculosis infection. Low molecular weight polypeptides (LMPs) and transporters with antigen processing (TAPs) are the main molecules in the processing and presentation pathway for intracellular antigens. This study was performed to elucidate whether these antigen-processing genes (LMP/TAP) polymorphisms could be associated with the risk of tuberculosis infection in China.

**Methodology/Principal Findings:**

We recruited 205 active pulmonary tuberculosis patients and 217 normal controls from Li population for this study. Four polymorphisms of LMP/TAP genes were determined by PCR-RFLP assay and haplotypes were constructed by software PHASE 1.0. Of the total four polymorphisms, genotype frequencies of LMP7 AA homozygote and CA heterozygote were significantly greater among cases compared to controls, with odds ratio of 3.77 (95% CI: 1.60–8.89; *P* = 0.002) and 2.97 (95% CI: 1.80–4.90; *P*<0.0001), respectively. The genotypes of TAP1-2 GG homozygote and AG heterozygote were more frequent in subjects with TB than in controls, with odds ratio of 3.94 (95% CI: 1.82–8.53; *P* = 0.001) and 2.87 (95% CI: 1.75–4.71; *P*<0.0001), respectively. Similarly, we found that haplotype B which carried LMP7 and TAP1-2 variations significantly increased the susceptibility to TB (OR = 3.674, 95% CI: 2.254–5.988; *P*<0.0001). Moreover, it is noteworthy that the homozygote of wild haplotype A (A/A) may be a strong protection for TB infection.

**Conclusions:**

Our findings suggested that LMP/TAP gene polymorphisms might be risk factors for TB infection among Li population in China.

## Introduction

Tuberculosis (TB), the leading cause of morbidity and mortality by a single infectious agent, is still a major health problem in the world. According to the annual report on global control of TB from WHO, about 9.4 million incident cases and 14 million prevalent cases occurred in 2009. Approximately 1.7 million people died of TB, including 0.38 million deaths among the HIV-positive people, and most cases were in the South-East Asia, African and Western Pacific regions (35%, 30% and 20%, respectively) [1].

It is well known that host genetic susceptibility, together with bacterial strains and environmental factors, plays an important role in determining TB predisposition and drug response. Only about 10% of the infected individuals develop the clinical disease while most infected people carry the bacteria without overt symptom [2], [3]. To date, many studies have shown evidence of association between host genetic polymorphisms and TB susceptibility, including CCL2/MCP-1, NRAMP1/SLC11A1, IRGM1, IL8, TLR, and NOD2 genes [4]–[9]. Most of these genes participate in immune response and their polymorphisms may lead to increase genetic susceptibility to TB.

The genes for low molecular weight polypeptides (LMPs) and transporters with antigen processing (TAPs) are located within the MHC class II region of chromosome 6 between the HLA DP and HLA DQ loci, and have been shown to play a critical role in the processing and presentation pathway for intracellular antigens [10]. The LMP genes encode two subunits which form a proteasome complex and mainly involve in the proteolysis of certain peptides [11]. The products of TAP genes compose a heterodimeric complex which translocates antigenic peptides from cytoplasm into the endoplasmic reticulum for binding the major histocompatibility complex (MHC) class I molecules for presentation to CD8+ cytotoxic T cells [12]. It has been reported that *Mycobacterium tuberculosis* (*Mtb*) protein presentation requires the cytosolic proteasomal degradation and subsequent TAP transportation [13], [14]. Although the mechanisms underlying the Class I presentation of *Mtb* antigens remain enigmatic, studies have demonstrated that it may use multiple processing pathways [15], [16]. In addition, a previous report also revealed that TAP-deficient mice exhibited an increased susceptibility to TB that was manifested by a decreased survival after infection, a greater bacterial burden, and more severe tissue pathology [17]. These data establish that MHC Class I antigen-processing pathway which requires cleavage of antigen peptides by LMP2/LMP7 and transportation of peptide fragments into the endoplasmic reticulum by TAP1/TAP2, is vital for controlling the *Mtb* infection and preventing the development of active TB.

Several polymorphisms within the coding region of LMP and TAP have been detected and are considered to be associated with a number of immune diseases, viral infection diseases, and even malignant tumors [18]–[22]. A few of these polymorphisms (TAP1 and TAP2) have been studied to determine predisposition towards TB in different ethnic groups, which have revealed the susceptibility to TB [23], [24]. The aim of this study was to investigate the associations between polymorphisms of LMP and TAP genes and susceptibility to active TB disease in Li population in Hainan province, an island of southern China in the South China Sea. The incidence of TB was 1.5 fold in Li population than in Han population [25]. Concurrently, we also attempted to determine whether the haplotypes covering these SNPs were linked to the development of TB and explored potential risk factors for TB.

## Methods

### Ethics Statement

This study was approved by the Institution Review Boards of Hainan Center for Disease Control and Prevention and the written informed consents were obtained.

### Subjects

Two hundred and five unrelated patients diagnosed with active pulmonary TB were enrolled in this study. These patients were followed up at Hainan Center for Disease Control and Prevention between 2005 and 2008. The diagnosis of pulmonary TB was based on the following criteria: 1) clinical symptom suggestive of TB; 2) chest radiographic evidence of active pulmonary TB in the upper lobes; 3) sputum smear positive with or without chest radiographic evidence of active pulmonary TB; 4) excluding the subjects with diseases such as lung carcinoma, pneumonia, diabetes and other immunosuppressive condition. Patients with the presence of all criteria were recruited.

The control group included 217 healthy unrelated adults without history of TB, autoimmune diseases, or other infectious diseases from Li population in Hainan province. The healthy volunteers were from the general population with the same socio-economic status and ethnic background as that of the patients, and were selected through the population survey of tuberculosis with PPD skin test results <5 mm and remaining uninfected during 2 years.

All subjects of TB cases and normal controls were HIV seronegative and BCG vaccinated. The demographic characteristics were described in Table 1.

**Table 1:** Demographic characteristics for tuberculosis cases and controls.

	Case n = 205	Control n = 217
gender		
male	138	105
female	67	112
age		
male	42.20±17.35	26.86±12.64
female	41.27±15.63	27.63±15.17
smoke		
male	43	30
female	2	2
drink		
male	45	32
female	3	1
BCG Vaccinated	Yes	Yes

### Genotyping analysis and haplotype construction

Genomic DNA was extracted from peripheral blood leukocytes of study subjects by standard procedures. Polymorphisms in LMP genes and TAP genes were genotyped using primer pairs as shown in Table 2. The four polymorphisms were detected by polymerase chain reaction (PCR) restriction fragment length polymorphism (RFLP) analysis, which abide by previously described methods [20]. The reaction was carried out in a 50 µl reaction mixture containing 4 µl of genomic DNA, 5 µl of 10×PCR buffer, 2.5 µl of 25 mM MgCl_2_, 1 µl of 10 µM dNTP, 1 µl of 20 µM each specific primers (shown in Table 1), 0.5 µl of 5 U Taq DNA polymerase, and 36 µl of ddH_2_O. The PCR was performed by denaturing at 94°C for 2 min, followed by thirty cycles as denaturation at 94°C for 40 s, annealing at 56.5°C (LMP2) and 57°C (LMP7, TAP1-1, TAP1-2) for 40 s, extension at 72°C for 40 s, and the final extension at 72°C for 10 min.

**Table 2:** Primer sets used for amplification and sequencing of LMP/TAP gene.

Gene Code position	variant	rs #	missense	PCR primers (sense/antisense)	Length (bp)	Annealing temperature
LMP2	C/T	rs17587	Arg→Cys	5′-CTCCACTTTACAGATGCAGA-3′	330	56.5°C
			(CGC→TGC)	5′-ACTTGGTGACTGTTGACTCC-3′		
TAP1-1	A/G	rs1057141	Ile→Val	5′-GCAGGTAACATCATGTCTCG-3′	430	57.0°C
			(ATC→GTC)	5′-GACAGATTGTGGGGAGAAGC-3′		
TAP1-2	A/G	rs1135216	Asp→Gly	5′-CAGTAGTCTTGCCTTTATCC-3′	405	57.0°C
			(GAC→GGC)	5′-ATGACTGCCTCACCTGTAAC-3′		
LMP7	C/A	rs2071543	Gln→Lys	5′-TCATGGCGCTACTAGATGTATG-3′	351	57.0°C
			(CAG→AAG)	5′-AACTCTTTGTCCTAACTTGCAC-3′		

The amplified products were purified and then digested using specific restriction endonuclease under the different conditions as per the manufacturer's instructions. After digestion, the fragments were electrophoresed in a 2% agarose gel and were visualized by ethidium bromide staining. The accuracy of genotyping was confirmed by direct sequencing of the random DNA samples (N = 10) from cases and controls for all polymorphic sites. Finally, the PHASE 1.0 software was used to construct the haplotypes for all the four gene polymorphisms [26].

### Statistical analysis

The chi-square test was used to compare genotype distribution and allele frequency in the TB patients and control subjects with SPSS 12.0 software packages. Hardy–Weinberg equilibrium was tested by Haploview 4.2 software. The haplotype frequencies were calculated with PHASE1.0 software. The associations between case and control groups were evaluated by utilizing the unconditional logistic regression model adjusted for gender, age, cigarettes smoking and alcohol drinking. Odds ratio (OR) and 95% confidence interval (95% CI) were estimated and a *P* value of <0.05 was considered to be significant.

## Results

### Characteristics of the Participants

The 205 patients with active pulmonary TB and 217 healthy controls with PPD skin test results <5 mm were studied. The demographic characteristics including age, gender, cigarettes smoking, and alcohol drinking were summarized in Table 1. Considering that all study subjects were BCG vaccinated, we only selected the non-infected individuals as control subjects in this preliminary study.

### Correlation of Genetic Polymorphisms and Tuberculosis

According to the previous studies of LMP/TAP gene polymorphisms of other diseases [20], [22], we selected four non-synonymous coding SNP. Genotypes (homozygote and heterozygote) were confirmed by using restriction fragment length polymorphism (RFLP) method according to different numbers of enzyme fragments. Table 2 listed the SNPs that were selected including their rs numbers and PCR amplification primers.

The genotype distributions for these four polymorphisms did not deviate significantly from Hardy-Weinberg equilibrium in both case and control groups. Table 3 listed the allele and genotype frequencies for all the polymorphisms. Allele frequencies for three polymorphism loci (TAP1-1/TAP1-2/LMP7) were revealed to have significant differences between the TB patients and controls (*P*<0.01). The genotype frequencies of LMP7 AA homozygote and CA heterozygote were significantly greater among cases compared to the healthy control group, with odds ratio of 3.77 (95% CI: 1.60–8.89; *P* = 0.002) and 2.97 (95% CI: 1.80–4.90; *P*<0.0001), respectively. Similarly, the genotype of TAP1-2 GG homozygote and AG heterozygote were more frequent in subjects with TB than in controls, with odds ratio of 3.94 (95% CI: 1.82–8.53; *P* = 0.001) and 2.87 (95% CI: 1.75–4.71; *P*<0.0001), respectively.

**Table 3:** Genotype, allele frequencies of the polymorphisms in the LMP and TAP genes.

Gene	Allele	Cases n (%)	Controls n (%)	*P* Value	Genetype	Cases n (%)	Controls n (%)	*P* Value*	OR*(95% CI)
LMP2									
	C	321 (78.29)	347 (79.95)		CC	124 (60.49)	135 (62.21)	—	
	T	89 (21.71)	87 (20.05)	0.553	CT	73 (35.61)	77 (35.48)	0.691	1.33 (0.33–5.33)
					TT	8 (3.90)	5 (2.30)	0.770	0.93 (0.58–1.49)
TAP1-1									
	A	299 (72.93)	352 (81.11)		AA	104 (50.73)	147 (67.74)	—	
	G	111 (27.07)	82 (18.89)	**0.005**	AG	91 (44.39)	58 (26.73)	**0.008**	**1.92** (1.19–3.12)
					GG	10 (4.88)	12 (5.33)	0.583	0.74 (0.25–2.18)
TAP1-2									
	A	240 (58.54)	322 (74.19)		AA	66 (32.20)	121 (55.76)	—	
	G	170 (41.46)	112 (25.81)	<**0.0001**	AG	108 (52.68)	80 (36.87)	<**0.0001**	**2.87** (1.75–4.71)
					GG	31 (15.12)	16 (7.37)	**0.001**	**3.94** (1.82–8.53)
LMP7									
	C	240 (58.54)	314 (72.35)		CC	59 (28.78)	110 (50.69)	—	
	A	170 (41.46)	120 (27.65)	<**0.0001**	CA	122 (59.51)	94 (43.32)	<**0.0001**	**2.97** (1.80–4.90)
					AA	24 (11.71)	13 (5.99)	**0.002**	**3.77** (1.60–8.89)

Significant p values are in bold.

* Logistic regression model, adjusted by gender, age, smoke and drink.

Of the other two polymorphisms, the prevalence of the TAP1-1 AG heterozygote was more significantly found in cases than in controls (OR = 1.92, 95% CI: 1.19–3.12, *P* = 0.008), while the frequency of GG homozygote had no considerable difference between the two groups (*P* = 0.583). In the LMP2 polymorphic site, no significant difference was observed in the distribution among cases and controls (*P*>0.05).

Considering the difference in gender distribution between case and control groups, we further analyzed our data by separating males and females. Similar results were obtained when samples were stratified by gender. Significant associations were observed in LMP7 and TAP1-2 polymorphism sites with either allelic or genotypic analysis (*P*<0.01). There was a little difference detected for TAP1-1 site in which male showed a trend of association but not in female group (Table 4). Therefore, the possible exerted confounding effect due to difference in gender distribution may be excluded.

**Table 4:** Genotype, allele frequencies and sex specific association analysis.

Gene and sex		n	Allele frequency (%)		*P* Value	Genotype frequency (%)			*P* Value*
LMP2			C	T		CC	CT	TT	
M	Case	138	213 (77.17)	63 (22.83)		81 (58.69)	51 (36.96)	6 (4.35)	
	Control	105	173 (82.38)	37 (17.62)	0.159	70 (66.67)	33 (31.43)	2 (1.90)	0.561
F	Case	67	108 (80.60)	26 (19.40)		43 (64.18)	22 (32.84)	2 (2.98)	
	Control	112	174 (77.68)	50 (22.32)	0.513	65 (58.03)	44 (39.29)	3 (2.68)	0.856
TAP1-1			A	G		AA	AG	GG	
M	Case	138	197 (71.38)	79 (28.62)		67 (48.55)	63 (45.65)	8 (5.80)	
	Control	105	178 (84.76)	32 (15.24)	**0.0005**	78 (74.29)	22 (20.95)	5 (4.76)	**0.014**
F	Case	67	102 (76.12)	32 (23.88)		37 (55.22)	28 (41.79)	2 (2.99)	
	Control	112	174 (77.68)	50 (22.32)	0.734	69 (61.61)	36 (32.14)	7 (6.25)	0.324
TAP1-2			A	G		AA	AG	GG	
M	Case	138	160 (57.97)	116 (42.03)		43 (31.16)	74 (53.62)	21 (15.22)	
	Control	105	158 (75.24)	52 (24.76)	<**0.0001**	62 (59.05)	34 (32.38)	9 (8.57)	<**0.0001**
F	Case	67	80 (59.70)	54 (40.30)		23 (34.33)	34 (50.75)	10 (14.92)	
	Control	112	164 (73.21)	60 (26.79)	**0.008**	59 (52.68)	46 (41.07)	7 (6.25)	**0.047**
LMP7			C	A		CC	CA	AA	
M	Case	138	158 (57.25)	118 (42.75)		38 (27.54)	82 (59.42)	18 (13.04)	
	Control	105	154 (73.33)	56 (26.67)	**0.0002**	55 (52.38)	44 (41.90)	6 (5.72)	**0.001**
F	Case	67	82 (61.19)	52 (38.81)		21 (31.34)	40 (59.70)	6 (8.96)	
	Control	112	160 (71.43)	64 (28.57)	**0.045**	55 (49.11)	50 (44.64)	7 (6.25)	**0.025**

Significant p values are in bold.

* Logistic regression model, adjusted by gender, age, smoke and drink.

### Association of Haplotype with Tuberculosis

To study the combined effects of the four polymorphic sites in LMP/TAP genes, we next carried out the haplotypic association analysis. The statistical software PHASE 1.0 was used to estimate differences in the haplotype frequencies, which calculated haplotype frequencies with an expectation maximization algorithm. A total of thirteen haplotypes (A-M) were constructed, and all of the haplotypes were found in both case and control groups. The distribution of different haplotypes in each group was summarized in Table 5.

**Table 5:** Haplotypes frequencies of the LMP/TAP genes in cases and controls.

Haplotypes	Loci[LMP2][TAP1-1][TAP1-2][LMP7]	Cases n (%)	Controls n (%)
Number of chromosome		410 (100)	434 (100)
A	CAAC	138 (33.66)	229 (52.76)
B	CAGA	97 (23.66)	52 (11.98)
C	TGAC	40 (9.76)	34 (7.83)
D	TAGA	9 (2.20)	26 (5.99)
E	CGGA	19 (4.63)	14 (3.23)
F	CAAA	15 (3.66)	18 (4.15)
G	CGAC	11 (2.68)	19 (4.38)
H	CGAA	23 (5.61)	7 (1.61)
I	CAGC	18 (4.39)	8 (1.84)
J	TGGC	18 (4.39)	8 (1.84)
K	TAAC	6 (1.46)	12 (2.76)
L	TAGC	9 (2.20)	4 (0.92)
M	TAAA	7 (1.71)	3 (0.69)

Among the thirteen haplotypes observed in the 205 TB patients and 217 controls recruited for the study, only three haplotypes represented at frequencies >5% (Table 5). These three haplotypes together accounted for >69.9% of the total haplotypes in both case and control groups. Thus, these haplotypes were selected to assess the susceptibility to TB (Table 6).

**Table 6:** Haplotypes distributions of the LMP/TAP genes in cases and controls.

Haplotypes	Case n	Control n
CAAC = A		
–/–a	67	68
–/A	138	69
A/A	0	**80**
CAGA = B		
–/–	108	166
–/B	97	50
B/B	0	1
TGAC = C		
–/–	165	184
–/C	40	32
C/C	0	1

a – denotes any haplotype, for example: –/A indicates the A haplotype in combination with any other haplotype.

We noticed these three haplotypes had relatively low frequencies with homozygote haplotypes, which may increase the inaccuracy of haplotype frequency estimation and lead to false-positive inference. Hence, we incorporated the homozygote and heterozygote of each haplotype and evaluated the association of global haplotype distributions with TB. The results showed that only haplotype B significantly associated with tuberculosis in our study population (OR = 3.674, 95% CI = 2.254–5.988, *P*<0.0001), as depicted in Table 7. No significant differences were observed in the other two haplotypes between the two groups.

**Table 7:** Distribution of haplotype frequencies in LMP/TAP genes among cases and controls.

Haplotypes	Case n (%)	Control n (%)	*P* Value^a^	OR (95% CI)
CAAC = A				
–/–b	67 (32.7)	68 (31.3)		----
–/A+A/A	138 (67.3)	149 (68.7)	0.867	0.960 (0.594–1.551)
CAGA = B				
–/–	108 (52.7)	166 (76.5)		----
–/B+B/B	97 (47.3)	51 (23.5)	**<0.0001**	**3.674** (2.254–5.988)
TGAC = C				
–/–	165 (80.5)	184 (84.8)		----
–/C+C/C	40 (19.5)	33 (15.2)	0.317	1.358 (0.745–2.474)

a Logistic regression model, adjusted by gender, age, smoke and drink.

b – denotes any haplotype, for example: –/A indicates the A haplotype in combination with any other haplotype.

## Discussion

As the second country with the largest number of incidence cases in the world, China is still facing great challenges to TB control. Though the incidence rate has slowly reduced from 99 (per 100 000 population) in 2005 to 96 (per 100 000 population) in 2009, the absolute number of cases continues to increase slightly from year to year [1]. Li population is the only minority nationality lived in Hainan province, with a total population of 1.28 million in 2010. The rate of smear-positive TB was 13.1% in Li population, compared to 9.9% in Han population [25]. Besides the socio-economic factors such as living condition, poverty, treatment delay and poor-quality care, host genetic susceptibility may be another significant risk factor for influencing TB incidence in different population.

In the present study, we focused on the association of polymorphic sites in LMP/TAP genes with TB susceptibility in Li population. We tested four polymorphisms in the coding regions of LMP2, LMP7 and TAP1 genes and observed all the polymorphisms in the subjects enrolled in our study. The polymorphism of LMP2 gene (rs17587) was reported as the substitution of arginine to histidine [27]. But we did not find histidine at this locus in any of the cases or controls. Instead, a cysteine replacement was observed in this population which was consistent with previous studies [20], [22]. These findings suggested that LMP and TAP polymorphisms may differ among populations as reported by other studies.

In our study, significant association between LMP/TAP genes and TB was observed when we compared our active TB patients and controls. The subjects containing LMP7 AA homozygote and CA heterozygote were found to be strongly associated with TB infection (OR = 3.77, 2.97, respectively). Similarly, the TAP1-2 polymorphism also exhibited a significant relation to TB infection. For the other two polymorphisms, no statistical significant associations were found at polymorphic sites of LMP2 and TAP1-1 genes to the risk of TB (Table 3). Similar to the individual SNPs, we also observed that haplotype B which carried LMP7 and TAP1-2 variations significantly increased the susceptibility to TB. Moreover, it is noteworthy that the homozygote of wild haplotype A (A/A) may be a strong protection on TB infection (Table 6). All these results indicated that the LMP7 and TAP1 gene polymorphisms might be risk factors for TB infection among Li population.

The critical roles of the LMP/TAP genes are consistent with the observed association for TB in our study. LMP is a cytosolic proteinase complex which hydrolyzes antigens into 8- or 9-residue peptides, and TAP is a member of the ATP-binding cassette transporter family which translocates antigenic fragments to MHC class I molecules in the endoplasmic reticulum. Previous studies have reported that LMPs proteasomes favor to promote cleavage of peptides after hydrophobic and basic residues but suppress cleavage after acidic residues. This effect results in the generation of peptides that preferentially ended with hydrophobic or basic carboxyl termini [28]. Furthermore, the polymorphisms located at TAP1/TAP2 gene coding region may affect the specificity of peptide presentation [29], [30]. Therefore, the interaction of LMP and TAP genes polymorphisms may subsequently interfere with the LMP/TAP-dependent translocation of disease associated peptides.

It is worth mentioning that the LMP and TAP genes are located close and centromeric to the HLA-DQB1 gene in chromosome 6 MHC class II region [31]. The MHC is the most gene-dense region of the human genome sequenced. It also encodes most polymorphic human proteins, some of which have over 200 allelic variants. Considering that strong linkage disequilibrium exists between HLA-DR and HLA-DQ genes, the strong associations for LMP7 and TAP1 genes might also be in linkage disequilibrium with HLA-DQB1 gene in our population. This hypothesis should be verified in the further study.

In 1997, Rajalingam R and colleagues first reported a statistical association between alleles in TAP2 region with pulmonary tuberculosis and tuberculoid leprosy susceptibility in the North India populations [23]. In contrast to our study, no significant differences in the prevalence of TAP1 alleles were observed. In addition, another recent study carried out in a Northwestern Colombian population also failed to detect any association of TAP1 gene with TB disease [24]. There may be several reasons for the discrepancy between our findings and those reported results. Recently, Stein took a systematic review from a genetic epidemiological perspective. She illustrated the key influencing factors for the inconsistency in TB literature, such as the phenotype definition both in cases and controls, population differences-more than just geography, complex genetic effects, and even potential global differences in *M. tuberculosis* strain [32]. Another study by Chang et al also mentioned that polymorphisms in different genes could affect antigen presentation to the same extent and therefore compensate for each other [33]. Our study was conducted in ethnically matched 205 active TB patients and 217 normal healthy controls recruited from the same population. Both the single SNP and the haplotype analyses supported the observed association of TB susceptibility. But on the other hand, there are also some limitations should be kept in mind. First, we couldn't differentiate the latent tuberculosis infection (LTBI) versus active TB disease, which previous studies have showed that some genes may differ between the two disease statuses [34]–[36]. Second, limited number of studies in the haplotype stratified analysis made the detection of small differences difficult. Third, although we analyzed our data by separating males and females and obtained similar results, the difference distribution in gender was still a major pitfall in this preliminary study. A comprehensive and systematic analysis with large sample sizes and two stage processes of TB infection (LTBI and TB) is still in progress.

In summary, our findings provide the first evidence of the association between LMP gene polymorphism and human susceptibility to TB disease. We also support the hypothesis that MHC class I-mediated antigen presentation may play an important role in the host defense to TB. Since the complexity and versatility of host immune response to *M. tuberculosis*, further studies with multiple genes interaction and multidisciplinary approaches including genomic, proteomic, and immunologic data, collectively, may better confirm the current findings.
